# Atopic dermatitis in the COVID-19 era: Results from a web-based survey

**DOI:** 10.1016/j.waojou.2021.100571

**Published:** 2021-08-24

**Authors:** Natalia Hernández, Gloria Sanclemente, Liliana Tamayo, Ángela López, Angela Seidel, Colombian Atopic Dermatitis Research Group Members, Natalia Hernandez, Gloria Sanclemente, Daniela Chaparro, Ángela López, Andrés Cortes, Ángela Seidel, Clara Inés Ortiz, Claudia Arenas, Esperanza Meléndez, Julio Amador, Liliana Tamayo, Lina Colmenares, María Claudia Guzmán, María Claudia Torres, Mariela Tavera, Mauricio Torres, Miriam Vargas, Mónica Novoa, Mónica Rivera, Natalia Vélez, Oscar Mora, Oscar Medina, Paola Cárdenas

**Affiliations:** f Dermatosoluciones SAS, Colombia; g Group of Investigative Dermatology (GRID), University of Antioquia, Medellin, Colombia; h Pontificia Universidad javeriana, Bogota, Colombia; i IPS Fototerapia Bojanini y López SAS, Colombia; j Centro Dermatológico Federico Lleras Acosta, Bogotá, Colombia; k Hospital Universidad del Norte, Barranquilla, Colombia; l Universidad Militar, Bogotá, Colombia; m Universidad Pontificia Bolivariana, Medellín, Colombia; n Universidad CES, Medellín, Colombia; o Fundación Ciencias de la Salud, Bogotá, Colombia; p Universidad del valle, Cali, Colombia; q Universidad CES, Medellín, Colombia; a Dermatosoluciones SAS, Location Address: Calle 90 # 19A 49 Cons 501, Bogotá, Colombia; b Group of Investigative Dermatology (GRID), University of Antioquia, Medellín, Colombia; c Universidad Pontificia Bolivariana, Medellín, Colombia; d IPS Fototerapia Bojanini y López SAS, Bogotá, Colombia; e Clínica Central del Quindío, Colombia

**Keywords:** Atopic dermatitis, COVID-19, Survey

## Abstract

Given that the COVID-19 era has changed the behavior of all individuals, and since previous reports about its possible impact on atopic dermatitis (AD) patients remained speculative, in this survey we aimed to explore the real impact of COVID-19 among AD patients.

All participants provided verbal consent prior to completing the survey. A 37-question web-based survey with no personal identifiers was sent to 212 previously identified AD patients. Itching, sleep disturbances, SARS-CoV-2, illness cost, economic dependence, monthly income, and monthly investment in AD before and during the pandemic, were all included in the analysis.

A response rate of 73.1% was obtained. The mean age of participants was 30 years-old, and 57% were women. Around 75% reported AD worsening, and 59.4% of the patients reported sleep problems. Uncertainty, anxiety, and pessimism were frequent during the pandemic. Only 1.3% tested positive for SARS-CoV-2, and it was only significantly associated with comorbidities (p=0.03; Chi^2^ Test). A significant difference was found in economic dependence and monthly income when compared between before and during the pandemic.

This study provides probably the best possible assessment of the clinical, social, and economic effects of the pandemic on patients with an already proven diagnosis of AD.

## Introduction

The acute respiratory distress syndrome associated with coronavirus 2 (SARS-CoV-2) also known as COVID-19, is an entity not previously identified in humans that began in Wuhan, China with the first case in December 2019, and which was declared a pandemic in March 2020.

Atopic dermatitis (AD) is a prevalent worldwide disease in children and adults that is influenced by genetics and environmental exposures that may lead to the triggering and/or flaring of the disease in predisposed individuals with an established epidermal barrier dysfunction.^1^, ^2^, ^3^ The COVID-19 might have affected AD patients,^4^ as isolation and vulnerability feelings generated by the pandemic may contribute to relevant psychological adverse events such as irritability, anxiety, emotional fatigue, anger, depression, and post-traumatic stress. Restrictive measures, smoking, a decrease in socioeconomic status, stress, frequent handwashing, and the use of antiseptic cleansers and hand sanitizers during these times, may also worsen the disease and can further contribute to skin damage.^5^, ^6^, ^7^ In addition, less exposure to sunlight that exerts an immunosuppressive effect on skin inflammation and prolonged wearing of facemasks expose patients to continuous pressure, friction, and heat and humidity trapping which in turn irritates the skin and worsens facial eczema.^8^

As the COVID-19 era has changed the behavior of all individuals, and since previous reports about its possible impact on AD patients remained speculative,^8^^,^^9^ in this survey we aimed to explore the real impact of COVID-19 among Colombian AD patients.

## Methods

All participants provided verbal consent prior to completing the survey. A 37-question web-based survey with no personal identifiers was sent to all 212 AD patients included in a previous cross-sectional in-person multicenter nationwide study of atopic dermatitis (manuscript in preparation) starting on June 13, 2020, and the survey was closed on September 5, 2020. Itching was assessed through a 0-10-point visual analogue scale and participants were asked about having sleep disturbances. Socioeconomic classification was stratified as: Stratum 1: Low-Low; Stratum 2: Low; Stratum 3: Medium-Low; Stratum 4: Medium; Stratum 5: Medium-High; Stratum 6: High, according to the Colombian National Administrative Department of Statistics (DANE).

All patients were asked if they had undergone a swab test for SARS-CoV-2. The cost of illness was determined according to patients' expended resources because of their disease needs. Data on economic dependence, monthly income, and monthly investment in AD before and during the pandemic, were all included in the analysis.

## Statistical analysis

A univariate analysis was performed to describe variables’ frequency. For continuous quantitative variables that fulfilled the assumption of normality (Kolmogorov Smirnov), the mean was used as a measure of central tendency and the standard deviation as a measure of dispersion. Absolute and relative frequencies and percentages were used for qualitative variables. Bivariate analysis was performed by the Chi-square test, or U of Mann Whitney, as appropriate. The significance level was specified at 0.05 for all tests. For data processing, the SPSS (Statistical Package for Social Sciences) version 27 was used.

## Results

One-hundred fifty-five patients responded to the survey (response rate: 73.1%). The population was made up mostly of women (57%), and the mean age of participants was 30 years-old age (Age range: 13–69 years). Regarding the economic characterization, around 50.3% of the patients belonged to the lower strata and 49.7% to higher strata.

With regard to health insurance, the majority of patients (78.8%) belonged to the contributory regime in which hospitalizations and physician visits and some prescriptions are covered by a health provider institution but non-prescription expenditures (ie, emollients and hypoallergenic or non-scented personal hygiene products) are not usually provided to patients, corresponding these to out-of-pocket expenses.

One-hundred sixteen patients out of 155 (75%) reported AD worsening with mild, moderate, and severe worsening in 70.3%, 18.7%, and 10.3% of patients, respectively.

The mean score of itch was 5.19, and 59.4% of the patients reported having sleep problems. About 7.7% of the population reported an increase in alcohol consumption, while smoking was increased in 3.9% of subjects. Of the 155 participants, 88 (56.8%), 85 (54.8%), 57 (36.8%), 43 (27.7%), 37 (23.9%), and 30 (19.4%) reported uncertainty, anxiety, pessimism, fear, depression, and attention deficit.

All AD patients remained on topical or systemic therapy. Of the 155 subjects, 25.8%, 23.9%, 21.9% 16.8%, and 9.7% were on topical betamethasone, tacrolimus, clobetasol, mometasone furoate, and desonide, respectively. In respect to oral antihistamines 12.8%, 9.7%, 7.7%, and 5.8% were on fexofenadine, loratadine, cetirizine, and hydroxyzine, respectively. Regarding systemic therapy, 18.7%, 5.8%, 5.8%, and 3.2% were on dupilumab, methotrexate, azathioprine, and oral steroids, respectively.

When the percentage of patients with AD flare were compared to the percentage of patients with comorbidities, psychiatric disorders, sleep disturbances, immunosuppressive therapy (prednisolone, methotrexate, azathioprine, and dupilumab) and SARS-CoV-2, no significant associations were found (All comparisons: p=>0.05; Chi^2^ Test).

The predominant skin areas involved were the face (47.7%), upper extremities (47.1%), lower extremities (40%), hands (36,8%), trunk (27,7%), and genitalia in 12.3% of the patients.

During the pandemic, 52.3% of the patients washed their hands 4–6 times per day, and 21.9% 7–10 times per day. Around 73.5% of the patients took 1 shower per day and 26.5% 2–3 showers per day. Only 30.3% of the patients used a syndet, 21.9% used regular soap, and 11% used antibacterial soap.

Around 85.4%, 7.1%, and 55.7% of the patients performed home duties with the previous use of emollients, cotton gloves, and disposable gloves, respectively.

Only 2/155 (1.3%) patient tested positive for SARS-CoV-2: one was asymptomatic and the other had lost of smell and taste, but no one was hospitalized or needed the intensive care unit (ICU).

Novel comorbidities were diagnosed during the pandemic in 13 patients (8.4%), and the most frequent was irritable bowel syndrome in 3 patients (1.9%), followed by sinusitis (1.29%) and migraine (1.29%).

When SARS-CoV-2 positive patients were compared to the percentage of patients with comorbidities, psychiatric disorders, sleep disturbances, immunosuppressive therapy (prednisolone, methotrexate, azathioprine, and dupilumab), and disease flare, there was a statistically significant association only with the presence of comorbidities (p=0.03; Chi^2^ Test).

Sixteen (10.3%) and 35 (22.6%) out of 155 patients changed their residency place and the cohabitants of their home during the pandemic, respectively. In addition, 11 patients (7.1%) lost their jobs, and monthly investment on disease during the pandemic was around 25, 50, and 100 US dollars in 25%, 36.1%, and 23% of the patients, respectively, and it did not vary in comparison to before the pandemic.

There were significant differences (p=0.00) when economic dependence and monthly income were compared in the periods before and during the pandemic.

## Discussion

Few studies have reported the impact of the COVID-19 pandemic on AD patients. This coronavirus disease has forced life changes in all populations and has been proposed to increase inflammatory skin diseases due to the modulation of neuroendocrine factors triggered by all the adverse psychological effects related with quarantine, diet changes, less physical activity, less exposure to sunlight, and exposure to indoor pollutants.^8^

As expected, the pandemic increased AD flares with mild worsening of the disease in the majority of our patients. Although itching remained moderate, a main concern was the presence of sleep disturbances, anxiety, and a sensation of uncertainty, which in turn could have influenced disease flaring. Other causes for disease worsening included increased hand washing, the lack of use of gentle soaps, and the prolonged use of gloves or masks which has been confirmed in this study, as main areas involved were the face, upper extremities, and hands.

A major concern of AD treating physicians during the early months of the pandemic was the risk of patients stopping emollients or prescribed medications due to the lockdown, supply limitations and the immunosuppressive effects of some systemic medications. Fortunately, all of our patients continued emollients and topical and systemic treatment without an increased risk of contracting COVID-19 or having major complications, as only 2 patients tested positive for SARS-CoV-2 and none required hospitalization. Such findings are in line with a report that was published 5 months after the initiation of the pandemic and which suggested that AD had a protective effect against COVID-19,^10^ as SARS-CoV-2 was not significantly associated with disease flaring, psychiatric disorders, sleep disturbances, or immunosuppressive therapy, which reinforces the guidance of tampering immunosuppressive medications and not discontinuing biologic therapy in AD patients unless the patient is > 60 years-old, has high risk comorbidities, or has symptomatic COVID-19.^11^^,^^12^ Interestingly, alcohol consumption and smoking during the outbreak was only slightly increased among our patients as has been reported in the general population in other web-based surveys.^13^ In addition, although testing positive for SARS-CoV-2 was significantly associated with comorbidities in this study, such a relationship has to be considered with caution, as these varied in type and were infrequent as a whole.

In this study there was a significant change in critical socioeconomic factors, from the onset of coronavirus and the months that followed, in our AD patients and/or their families. Although our results showed that few individuals have lost their jobs and were forced to change residencies and in-house cohabitants, it should be considered that a significant percentage of patients began to depend economically on someone else, and the majority of these households reduced their monthly income despite the fact that they had to continue spending resources on AD treatment.

Among the study limitations was the probable sample bias that may have led to the selection of participants for internet coverage and the observational nature of the study, as causal relations between variables could not be confirmed. Another limitation was that due to restrictions imposed by our government during the lock-down phase of the pandemic, we were unable to perform in-person evaluations of AD severity, and AD flaring had to be assessed by asking patients if they felt better or worse during the quarantine months. However, this study provides probably the best possible assessment of the clinical, social, and economic effects of the pandemic on patients with an already proven diagnosis of AD.

## Abbreviations

SARS-2: Acute respiratory distress syndrome associated with coronavirus 2; AD: Atopic dermatitis.

## Funding

Not applicable.

## Availability of data and materials

Data and materials are sent as an excel database.

## Authors contributions

Natalia Hernandez: Conceptualization, acquisition of data, writing-original draft preparation.

Gloria Sanclemente: Conception and design of the study, methodology, acquisition of data, original and draft preparation, analysis and interpretation of data, writing-reviewing and editing of the manuscript.

Liliana Tamayo: Acquisition of data, writing-original draft preparation.

Angela Lopez: Acquisition of data, writing-original draft preparation.

Angela Seidel: Acquisition of data, writing-original draft preparation.

## Ethical statement

All authors assure that in this study the following has been fulfilled:

1) This material is the authors' own original work, which has not been previously published elsewhere.
2) The paper is not currently being considered for publication elsewhere.
3) The paper reflects the authors' own research and analysis in a truthful and complete manner.
4) The paper properly credits the meaningful contributions of co-authors and co-researchers.
5) The results are appropriately placed in the context of prior and existing research.
6) All sources used are properly disclosed and cited.
7) All authors have been personally and actively involved in substantial work leading to the paper, and will take public responsibility for its content.

The project in which this survey is framed (Title: “Carga de la Enfermedad y Calidad de Vida de Pacientes Adultos y Adolescentes con Eccema Atópico: Caracterización Clínica y Sociodemográfica” and its informed consent were both approved by the Ethics and Investigation Committee of the IPS Universitaria as stated in Act # 134 of May 29, 2019.

## Authors' consent for publication

All authors have consented to the publication of this study.

## Declaration of competing interest

Patients from this study are participating in a project sponsored by the Asociación Colombiana de Dermatología y Cirugía Dermatológica (Asocolderma) through a 10.13039/100004339Sanofi Laboratories grant. However, this pharmaceutical laboratory has not participated in any workshop related to the study and has not being involved in the analysis or design and development of the study.
